# Synchronous mucosal Schwann-cell hamartomas in a young adult suggestive of mucosal Schwann-cell harmatomatosis: a case report

**DOI:** 10.1186/s12876-015-0349-4

**Published:** 2015-10-06

**Authors:** Jeong Mo Bae, Joon Young Lee, Junhun Cho, Sang Ah Lim, Gyeong Hoon Kang

**Affiliations:** 1 Department of Pathology, The Armed Forces Capital Hospital, 81, Saemaeul-ro 177beon-gil, Bundang-gu, Seongnam-si, Gyeonggi-do 463-040 South Korea; 2 Department of Internal Medicine, The Armed Forces Capital Hospital, 81, Saemaeul-ro 177beon-gil, Bundang-gu, Seongnam-si, Gyeonggi-do 463-040 South Korea; 3 Department of Pathology, Seoul National University College of Medicine, 28 Yongon-dong, Chongno-gu, Seoul, 110-744 South Korea; 4 Division of Gastroenterology, Department of Internal Medicine, Korea University Kuro Hospital, 148, Gurodong-ro, Guro-gu, Seoul, 152-703 South Korea; 5 Department of Internal Medicine, Korea University College of Medicine, Seoul, South Korea

**Keywords:** Schwann cells, Hamartomas, Colon polyps, Ganglioneuroma

## Abstract

**Background:**

Mucosal Schwann-cell hamartoma is a rare mesenchymal polyp that presents in the intestine. Despite lacking ganglion cells, it resembles a gastrointestinal ganglioneuroma.

**Case presentation:**

We report a case of synchronous mucosal Schwann-cell hamartomas in a young male patient, who presented with a single discrete polyp in the mid-rectum and multiple polypoid mucosal lesions in the distal rectum.

**Conclusion:**

To the best of our knowledge, this is the first report of a case of multiple mucosal Schwann-cell hamartomas.

## Background

Colonoscopy is the most effective tool for detecting colorectal cancers and colorectal polyps [1]. Most colorectal polyps, including conventional adenomas (e.g., tubular, villous, and tubulovillous adenomas) and serrated polyps (e.g., hyperplastic polyps, sessile serrated adenomas, and traditional serrated adenomas), are premalignant epithelial polyps. Mesenchymal polyps that protrude into the intestinal lumen are rare; however, their detection rate can be improved with screening colonoscopy. Based on histomorphology and cells of origin, intestinal mesenchymal polyps are classified into various categories, including gastrointestinal stromal tumors, smooth muscle tumors, lipogenic tumors, and neural tumors.

Gastrointestinal neural tumors include ganglioneuromas, perineuromas, schwannomas and neurofibromas. In 2009, Gibson et al. reported 26 cases of colorectal polyps that demonstrated pure Schwann cell proliferation in the lamina propria not associated with neurofibromatosis type 1 (NF1), and designated these polyps as mucosal Schwann-cell hamartomas [2]. Based on histomorphology, mucosal Schwann-cell hamartomas can be distinguished by schwannomas which have Verocay bodies, Antoni A and Antoni B areas and lymphoid cuffs. Neurofibromas can be distinguished by the presence of fibroblasts, perineurial-like cells and axons, and ganglioneuromas can be distinguished by the presence of ganglion cells. Although multiple polyps can develop in certain gastrointestinal neural tumors, such as neurofibromas and ganglioneuromas, it is unclear whether mucosal Schwann-cell hamartomas are associated with multiple polyps [2].

In this report, we describe a case of synchronous mucosal Schwann-cell hamartomas in a young adult, who presented with a single discrete polyp in the mid-rectum and multiple polyposis-like mucosal lesion in the distal rectum. We finally suggest multiple mucosal Schwann-cell hamartomas or mucosal Schwann-cell hamartomatosis as the possible diagnosis.

## Case presentation

**Fig. 1:**
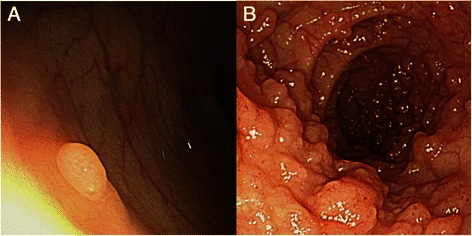
Proctoscopic findings. **a**. A 4-mm sized polyp in the mid-rectum, **b**. A polyposis-like mucosal lesion in the distal rectum

**Fig. 2:**
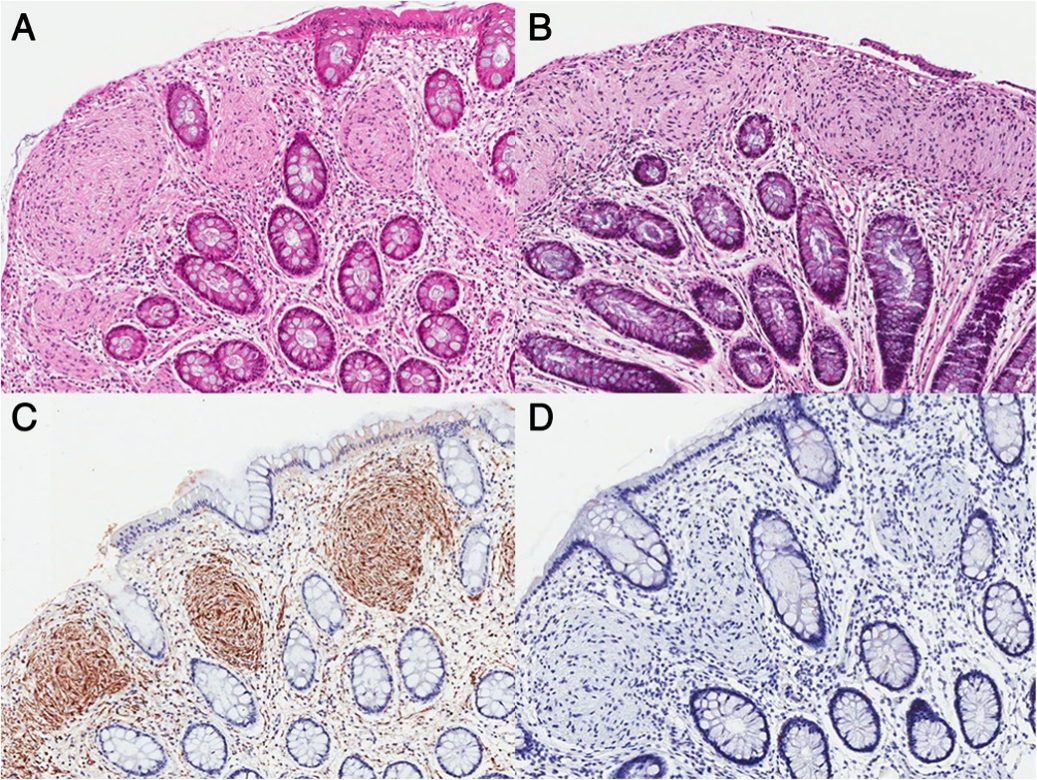
Histologic findings of mucosal Schwann-cell hamartomas. **a**. A polyp in the mid-rectum (H&E, x40), **b**. A polyposis-like mucosal lesion in the distal rectum (H&E, x40), **c**. Strong positive immunoreactivity for S-100 (x40), **d**. Negativity for synaptophysin (x40)

A 20-year-old man visited the Armed Forces Capital Hospital with symptoms of abdominal discomfort and loose stools. His mother had a history of early stage colorectal cancer and his grandfather died of colorectal cancer. Physical examination of the head, neck, lungs, heart, skin and other systems was unremarkable. He had normal bowel sounds with no abdominal tenderness or palpable abdominal mass. On procto-colonoscopic examination, a 4-mm sized polyp was observed in the mid-rectum (Fig. 1a), and scattered tiny polyp-like mucosal elevations were observed in the distal rectum (Fig. 1b). The polyp in the mid-rectum was removed by biopsy forceps, and three pieces of rectal mucosa in the distal rectum were randomly sampled. On microscopic examination, the polyp in the mid-rectum and one of the three pieces obtained from the distal rectum showed plexiform proliferation of spindle cells in the lamina propria, intervening the adjacent crypts. In addition, microscopy revealed that all cells were spindle-shaped and had elongated tapered nuclei, amphophilic to eosinophilic cytoplasm with indistinct cell borders, and no nuclear atypia, pleomorphism or mitoses. On immunohistochemical analysis, the lesions showed diffuse strong positivity for S-100 protein and no activity for CD117, CD34, epithelial membrane antigen (EMA), smooth muscle actin, and synaptophysin (Fig. 2). Neurofilament protein (NFP) stain showed no positive axon. We sequenced all exons of the *NF1* and *RET* genes using DNA derived from the blood leukocytes of the patient and found no evidence of a disease-causing mutation. To rule out *NF1* mosaicism, we performed targeted resequencing (Ion Ampliseq™ Comprehensive Cancer Panel, Life technologies®) of *NF1* genes using DNA extracted from formalin-fixed paraffin-embedded tissues of the lesion, and found no disease-causing mutation in *NF1* gene of the lesion. Considering the histological findings and the immunohistochemical results, we diagnosed these polyps as mucosal Schwann-cell hamartomas.

## Conclusion

Intestinal polyps containing neural proliferations in the lamina propria and lacking ganglion cells have been referred as “neuromas” or “neurofibromas”. However, some of these polyps are solely composed of S-100-positive Schwann cells, which distinguish them from true “neuromas” and “neurofibromas”. In 2009, Gibson et al. named these lesions “mucosal Schwann-cell hamartomas”[2]. Since then, only a few case reports have described these lesions [3–5]. Notably, there is limited literature on characterization of clinical features of mucosal neural proliferative lesions as these lesions are rare and only incidentally found [6]. In 2013, Bae et al. reported a case of mucosal Schwann-cell hamartoma in a 41-year-old woman and provided a literature review of 32 cases of mucosal Schwann-cell hamartomas [7]. The median age of the 32 patients at diagnosis of mucosal Schwann-cell hamartomas was 59 (range: 34 – 88) and the male to female ratio was 0.68:1 (13 males and 19 females). Mucosal Schwann-cell hamartomas were more frequent in the distal colorectum relative to the splenic flexure than in the proximal colon – in 32 cases reviewed, lesions were in the proximal colon in six cases and in the distal colorectum in the remaining 26 cases. Most reported cases of mucosal Schwann-cell hamartomas developed as a single polyp. In fact, it is not known whether mucosal Schwann-cell hamartoma can develop into multiple/diffuse polyps.

Mucosal Schwann-cell hamartomas are composed of uniform, bland spindle cells with elongated, tapering, or wavy nuclei, abundant dense cytoplasm, and indistinct cell borders. These cells entrap the colonic crypts without whirling, palisading, or fascicular architecture. These lesions show diffuse positivity for S-100 and NFP stain often shows rare axons. In fact, a diagnosis of mucosal Schwann-cell hamartomas should be made by excluding resembling lesions by careful histologic examination. Neurofibromas consist of heterogeneous cellular compositions, including Schwann cells, fibroblasts, perineurial-like cells and NFP-positive scattered axons [2]. Mucosal neuromas consist of disorganized and tortuous nerve bundles surrounded by a thickened perineurium that is positive for EMA [8]. Ganglioneuromas are composed of ganglion cells, nerve fibers, and Schwann cells [2].

Certain types of intestinal neural tumors develop as multiple or diffuse polypoid lesions in the context of inherited syndromes. Gastrointestinal neurofibromas have a strong association with NF1; however, a few sporadic intestinal neurofibromas have also been reported [9]. Diagnosis of NF1 can be based on clinical diagnostic criteria and confirmation of germline *NF1* mutation. Detection of *NF1* mutation may be challenging due to the large size of *NF1* gene and the lack of hotspot mutation [10]. Sanger sequencing can detect 88.8% of *NF1* mutation, whereas ancillary methods such as multiplex ligation-dependent probe amplification and targeted next generation sequencing improve the detection rate to 97% [10]. Mucosal neuromas are highly associated with multiple endocrine neoplasia type 2b (MEN-2b), which occurs in patients with germline mutation of *RET* genes [11]. Intestinal ganglioneuromatous polyposis and diffuse ganglioneuromatosis affect individuals with familial adenomatous polyposis, Cowden syndrome, tuberous sclerosis, NF1, MEN-2b and juvenile polyposis [6].

In this report, we presented a case of synchronous mucosal Schwann-cell hamartomas located in the mid-rectum and in the distal rectum of a young adult. We were unable to inspect the entire polypoid mucosal lesion in the distal rectum in this patient because of the benign nature of mucosal Schwann-cell hamartomas. Although we cannot exclude the possibility of ganglion cells in the remaining areas of the polypoid mucosa, we confirmed the absence of ganglion cells in the specimens by immunohistochemical analysis. In addition, we confirmed the absence of germline mutations in the *NF1* gene and in the *RET* gene by Sanger sequencing of leukocyte DNA and the absence of *NF1* mosaicism by targeted resequencing of the lesion. To the best of our knowledge, this is the first report of a mucosal Schwann-cell hamartoma in an adult under 30 years of age and also of a case of synchronous mucosal Schwann-cell hamartomas. Particularly, we suggest multiple mucosal Schwann cell hamartomas or mucosal Schwann-cell hamartomatosis as the likely diagnosis.

In summary, we presented a case of synchronous mucosal Schwann-cell hamartomas in a young adult and suggested multiple mucosal Schwann-cell hamartomas or mucosal Schwann-cell hamartomatosis as the possible diagnosis.

## Consent

Written informed consent was obtained from the patient for publication of this case report and any accompanying images. A copy of the written consent is available for review by the Editor in Chief of this journal.
